#  Randomized Trial of 2 Schedules of Meningococcal B Vaccine in Adolescents and Young Adults, Canada^1^

**DOI:** 10.3201/eid2603.190160

**Published:** 2020-03

**Authors:** Joanne M. Langley, Soren Gantt, Caroline Quach, Julie A. Bettinger, Scott A. Halperin, Jill Mutch, Shelly A. McNeil, Brian J. Ward, Donna MacKinnon-Cameron, Lingyun Ye, Kim Marty, David Scheifele, Erin Brown, Joenel Alcantara, The Canadian Immunization Research Network

**Affiliations:** Canadian Center for Vaccinology, Dalhousie University, IWK Health Centre, and Nova Scotia Health Authority, Halifax, Nova Scotia, Canada (J.M. Langley, S.A. Halperin, J. Mutch, S.A. McNeil, D. MacKinnon-Cameron, L. Ye);; Vaccine Evaluation Center, University of British Columbia, Vancouver, British Columbia, Canada (S. Gantt, J.A. Bettinger, K. Marty, D. Scheifele);; University of Montreal, Montreal, Quebec, Canada (C. Quach);; Research Institute of the McGill University Health Centre, Montreal, Quebec, Canada (C. Quach, B.J. Ward);; University of Calgary, Calgary, Alberta, Canada (E. Brown, J. Alcantara)

**Keywords:** Neisseria meningitidis serogroup B, meningococcus serogroup B, meningitis/encephalitis, disease outbreaks, prevention, vaccines, humans, student health services, meningococcal vaccine, 4C-MenB, bacteria, Canada, adolescents, young adults

## Abstract

Emergency vaccination programs often are needed to control outbreaks of meningococcal disease caused by *Neisseria meningitidis* serogroup B (MenB) on college campuses. Such campaigns expend multiple campus and public health resources. We conducted a randomized, controlled, multicenter, observer-blinded trial comparing immunogenicity and tolerability of an accelerated vaccine schedule of 0 and 21 days to a longer interval of 0 and 60 days for 4-component MenB vaccine (MenB-4C) in students 17–25 years of age. At day 21 after the first MenB-4C dose, we observed protective human serum bactericidal titers >4 to MenB strains 5/99, H44/76, and NZ 98/254 in 98%–100% of participants. Geometric mean titers increased >22-fold over baseline. At day 180, >95% of participants sustained protective titers regardless of their vaccine schedule. The most common adverse event was injection site pain. An accelerated MenB-4C immunization schedule could be considered for rapid control of campus outbreaks.

Campus outbreaks of meningococcal disease caused by *Neisseria meningitidis* serogroup B (MenB) are rare, but case-fatality rates are 5.3%–10.0%, and 10%–20% of survivors have long-term health effects (*1*). In addition, MenB outbreaks cause public distress and anxiety (*2*). Enhanced person-to-person transmission among young persons living in close quarters and having close social contacts on college campuses is thought to increase risk of outbreaks (*3*). During 2008–2017, a total of 12 campus-based clusters of MenB occurred at residential universities in North America (*4*), 11 in the United States (*4*) and 1 in Canada (*5*).

The public health response to a MenB outbreak includes education about prevention and early recognition of disease, antimicrobial drug prophylaxis for close contacts, and either preexposure or early postexposure vaccination (*3*). Two MenB vaccines are now available, a 4-component protein-based vaccine (MenB-4C [Bexsero; GlaxoSmithKline, https://www.gsk.com]) and a bivalent factor H binding protein-based vaccine (MenB-FHbp [Trumenba; Pfizer Inc., https://www.pfizer.com]). In Canada, MenB-4C was approved for use in 2013 for persons 2 months–17 years of age. MenB-4C is given as 2 doses >1 month apart (*6*). MenB-FHbp was authorized in 2017 and is given as 3 doses at 0, 1–2, and 6 months, or 2 doses 6 months apart. During MenB outbreaks, MenB-4C has been administered in 2 doses at varying schedules, including 0 and 30 days, 0 and 6–8 weeks, 0 and 2 months, and 0 and 10 weeks (*7*–*9*). However, few controlled studies have investigated the immunogenicity and reactogenicity of MenB-4C in older adolescents and young adults to compare various vaccination schedules (*10*–*13*).

Management of organization-based outbreaks on college campuses demands considerable resources (*3*,*14*), and disease transmission must be interrupted quickly. A shorter dosing schedule might provide more rapid individual direct protection and be easier to schedule around exams and school breaks. When MenB-4C vaccine was authorized in Canada, public health stakeholders identified a need to assess shorter dosing schedules for outbreak control to reduce the strain on public health resources by implementing the vaccine campaign in a single condensed period instead of 2 separate deployments. In a study supported by the Canadian Immunization Research Network (CIRN), we compared immunogenicity and tolerability of an accelerated MenB-4C vaccine schedule of 2 doses at 0 and 21 days to a longer interval of 0 and 60 days to facilitate outbreak control.

## Methods

We conducted a randomized, 1:1, observer-blind, controlled clinical trial to evaluate reactogenicity and immunogenicity of MenB-4C in 2 different vaccine schedules in Canada. We enrolled participants in Halifax, Nova Scotia; Montreal, Quebec; and Vancouver, British Columbia, during September 21–October 27, 2015. The last participant completed the final visit, day 180, on April 28, 2016.

The study was performed in compliance with Good Clinical Practice guidelines ICH E6 (R2), the World Medical Association Declaration of Helsinki (https://www.wma.net), and national regulatory requirements of Canada. The study protocol was approved by local institutional review boards in each city. The trial is registered at ClinicalTrials.gov (https://clinicaltrials.gov) under NCT02583412.

### Participants

We recruited study volunteers by advertising through social and traditional media on university campuses and through electronic mail to distribution lists of each study site. Eligible participants were 17–25 years of age, in good health, and attending school or university during the 2015–16 academic year. Volunteers were excluded if they were pregnant, planning pregnancy during the study period, or lactating; if they had a known medical illness, immunodeficiency, or autoimmune disease; if they previously received a MenB vaccine or had bacteriologically confirmed *N. meningitidis* disease; or if they had hypersensitivity to any components of vaccine products of MenB-4C or hepatitis A vaccine (HAV) used in the study.

### Vaccines

MenB-4C contains 4 *N. meningitidis* protein antigens adsorbed on aluminum hydroxide: factor H binding protein (fHbp), Neisserial adhesin A (NadA), Neisserial heparin binding antigen (NHBA), and Porin A P1.4 (PorA) from outer membrane vesicles. MenB-4C is provided in a sterile suspension for intramuscular injection of 0.5 mL/dose (*6*). For control, we used the HAV vaccine Havrix (GlaxoSmithKline Inc.). Havrix contains formaldehyde-inactivated hepatitis A virus adsorbed onto aluminum hydroxide and is provided in a sterile suspension for intramuscular injection in the deltoid of 1.0 mL/dose for this age group (*15*). Both vaccines are stored at 2°C–8°C. The first dose was given in the nondominant arm and the second dose was given in the contralateral arm.

### Study Procedures

Participants went to the study site on days 0, 21, 42, 60, 81, and 180. After the study nurse determined eligibility, obtained signed informed consent, and collected demographic characteristics on day 0, participants were randomly allocated 1:1 to the accelerated or longer intervals in blocks of 4 by site. On day 0, all participants received an unblinded first dose of MenB-4C vaccine. On day 21, participants received a second, blinded injection of MenB-4C if they were in the accelerated vaccine schedule group or HAV if they were in the longer interval group. On day 60, the accelerated schedule participants received HAV and the longer interval participants received MenB-4C. Study nurses drew 13 mL of blood at each visit to evaluate human serum bactericidal assay (hSBA) and factor H responses. 

At days 21 and 60, an unblinded nurse concealed vaccine identity from participants by covering the vaccine label, laying an opaque cloth over the administration tray, and prohibiting anyone except the unblinded nurse in the room with the participant for vaccine administration. The study participant was not told whether the vaccination was MenB-4C or HAV at the time of the observer-blinded injections. Participants who had not completed a prior 2-dose series of HAV were offered the second dose beginning at day 236.

### Outcome Measures

Our primary objective was to measure protective immune responses to the MenB vaccine by using hSBA titers against 3 meningococcal strains: strain New Zealand (NZ) 98/254 for the PorA P1.4 antigen of outer membrane vesicles, strain 5/99 for NadA, and strain H44/76 for fHbp. We did not evaluate the immune response to the NHBA component of the vaccine because a suitable indicator strain that matches this component is not available. The US Food and Drug Administration noted the unavailability of a suitable strain for assessing bactericidal activity of NHBA-specific antibodies in its licensing correspondence (*16*).

Dominique A. Caugant of the World Health Organization Collaborating Centre for Reference and Research on Meningococci at the Norwegian Institute of Public Health provided *N. meningitidis* serogroup B strains NZ 98/254, 5/99, and H44/76. The Alberta Children’s Hospital Research Institute of the University of Calgary performed hSBA by using standard methods (*17*) and human serum as a complement source. Laboratory staff were unaware of study group assignment. We assessed immune responses before MenB-4C vaccine and on days 21, 42, 81, and 180.

Our secondary objective was to compare the reactogenicity of MenB-4C given in an accelerated schedule to dosing on a longer interval. Study participants reported on solicited local adverse events (AE), such as redness, swelling, and pain, and on systemic AEs, including fever, drowsiness, nausea, diarrhea, vomiting, and generalized muscle aches. Participants recorded severity of AEs 0–6 days after each vaccination on a memory aid provided by study nurses (Appendix Table 1). Participants also recorded unsolicited AEs 0–21 days after each vaccination on a memory aid. Serious AEs were collected throughout the study.

We selected the sample size of 60 participants/study group on the basis of feasibility. Previous studies of MenB-4C in adolescents indicated that high bactericidal activity was likely after 1 vaccine dose (*18*). By using a margin of noninferiority of 0.15, and assuming that the probability of achieving a 1:4 hSBA titer with the standard schedule was 0.95, we estimated a sample size of 60 participants/group to provide a power of >0.76 to declare noninferiority, if the probability of 1:4 activity with the accelerated schedule was >0.91.

We considered all participants who received >1 dose of vaccine part of the total vaccinated cohort (TVC). We also had an according to protocol (ATP) cohort, which included all participants in the TVC who met the inclusion criteria and received vaccination according to the administration route and vaccination site of the protocol through the end of the study.

We assessed baseline comparability of treatment groups by using binomial estimates and Fisher exact test for binary variables and Student *t*-tests and CIs for continuous variables. We analyzed continuous variables of point estimates and interval estimates for means, and assessed differences between groups by using Student *t*-test and analysis of variance.

We calculated geometric mean titers (GMTs) for hSBA and 2-sided 95% CIs by group. We calculated hSBA results by using the proportion of participants at each serologic point with a titer >1:4, which is considered protective against each MenB strain (*19*). We did not stratify the analysis by baseline titer.

We used the Fisher exact test to assess differences in rates of solicited adverse events between treatment groups. We performed all statistical analyses by using 2-sided tests with a type I error of 5%. We did not include missing values in the analyses, imputation of missing values, or adjustments for multiple comparisons. We used a secure, internet-based data management platform (Dacima Software, https://www.dacimasoftware.com) to assemble study data and SAS version 9.4 or higher (SAS Institute, Inc., https://www.sas.com) to conduct analyses. 

## Results

**Figure 1:**
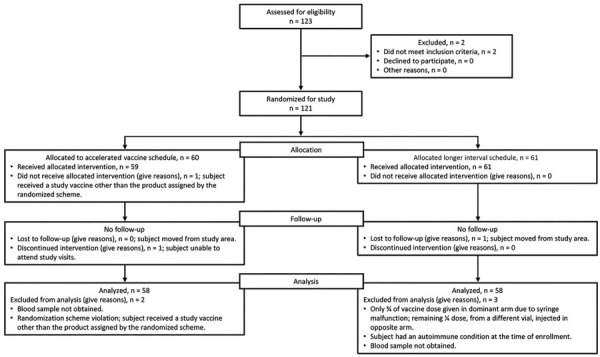
Flowchart of participant inclusion and follow-up for a trial of 4-component protein-based meningococcal B vaccine, Canada.

Among 121 participants, we had a retention rate of 99% (119/121); 2 participants did not complete follow-up. We had 116 participants the in ATP cohort and we analyzed results from 58 participants in each vaccination group (Figure 1).

Of 121 participants, 84 were female and 37 male. We allocated 60 participants to the accelerated vaccine group and 61 to the longer interval group according to the study protocol. The mean age for participants was 21.4 years. Participant’s body mass index, tobacco exposure, concomitant medication use, concurrent health conditions, and previous receipt of nonserogroup B meningococcal vaccines were similar between the 2 study groups (Table 1). 

**Table 1:** Characteristics of study participants in trial of MenB-4C vaccine, Canada*

Characteristic	Study groups		Total	p value
	Accelerated	Longer interval		
Mean age, y (SD)	21.2 (2.9)	20.7 (2.9)	20.6 (2.9)	0.44
Age range, y†	17–26	1 –26	17– 6	
Sex, no. (%)				
M	18 (30)	19 (31.1)	37 (30.6)	1.0
F	42 (70)	42 (68.9)	84 (69.4)	
Body mass index, median	23	22.8	22.9	0.17
Concurrent health conditions, no. (%)‡	29 (48.3)	49 (30)	59 (48.8)	1.0
Concomitant medication, no. (%)§	50 (83.3)	52 (85.2)	102 (84.3)	0.8
Tobacco use, no. (%)	9 (15)	8 (13.1)	17 (14.1)	0.79
Prior receipt of non-Men B vaccine, no. (%)#	49 (90.7)	46 (90.2)	95 (90.5)	1.0

*The accelerated schedule was MenB-4C vaccine at 0 and 21 days. The longer interval schedule was MenB-4C vaccine at 0 and 60 days. MenB, *Neisseria meningitidis* serotype B; MenB-4C, 4-component protein-based menB. †The first day of the birth month used to calculate age for inclusion criteria of 17–25 years of age, ‡The most common medical conditions were asthma, migraine headaches, seasonal allergies, and musculoskeletal complaints. §The most common concomitant medications were annual influenza vaccine, contraceptive medications, over-the-counter pain and cold preparations, and asthma and allergy medications.  #In Canada, the public health vaccine program includes a monovalent meningococcal C conjugate vaccine at 12 months of age and quadrivalent or monovalent meningococcal C conjugate vaccine in adolescence.

### Immunogenicity

**Figure 2:**
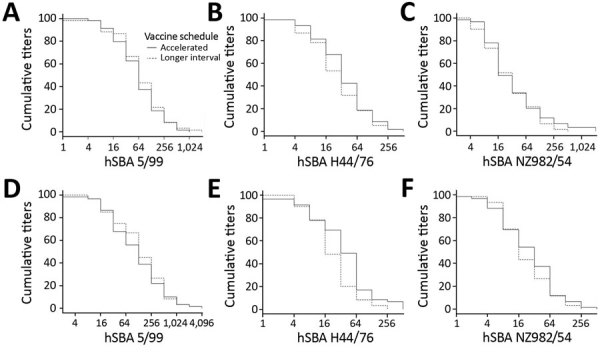
Reverse cumulative distribution curves of hSBA titers to 3 vaccine strains in recipients in trial of 4-component protein-based meningococcal B vaccine administered at 0 and 21 days compared with 0 and 60 days, Canada. A), B), and C) Comparisons made at day 21. D), E), and F) Comparisons made at day 180. hSBA, human serum bactericidal antibody; hSBA 5/99, Neisserial adhesin A surface proteins; hSBA H44/76, factor H binding protein; hSBA 982/54, New Zealand outer membrane vesicle.

At baseline, participants had hSBA titers against all 3 strains, but titers were lowest for strain H44/76 (>20.7%) and highest for strain 5/99 (<89.7%). Titers rose quickly after the first MenB-4C dose; 98%–100% of participants had titers ≥1:4 for all 3 antigens by day 21 (Table 2; Figure 2). The lower limit of the 95% CI for any hSBA strain at day 21 was 90.8% (Table 2). Titers remained high in >95% of participants in both study groups at day 180. 

**Table 2:** Percentage of participants with hSBA titers >1:4 by strain in a trial of MenB-4C vaccine from day 0 to day 180, Canada*

Meningococcal strain	Participants with hSBA, % (95% CI)				
	Day 0	Day 21	Day 42	Day 81	Day 180
5/99					
Accelerated	67.2 (53.7–79.0)	100 (93.8–100)	100 (93.7–100)	100 (93.7–100)	100 (93.7–100)
Longer interval	89.7 (78.8–96.1)	98.3 (90.8–100)	98.3 (90.8–100)	100 (93.8–100)	100 (93.7–100)
H44/76					
Accelerated	20.7 (11.2–33.4)	98.3 (90.8–100)	98.2 (90.6–100)	98.2 (90.6–100)	96.5 (87.9–99.6)
Longer interval	43.1 (30.2–56.8)	98.3 (90.8–100)	96.6 (88.1–99.6)	100 (93.8–100)	100 (93.7–100)
NZ98/254					
Accelerated	51.7 (38.2–65.0)	98.3 (90.8–100)	100 (93.7–100)	100 (93.7–100)	98.2 (90.6–100)
Longer interval	65.5 (51.9–77.5)	100 (93.8–100)	100 (93.8–100)	100 (93.8–100)	98.2 (90.6–100)

*The accelerated schedule was MenB-4C vaccine at 0 and 21 days. The longer interval schedule was MenB-4C vaccine at 0 and 60 days. 5/99, Neisserial adhesin A surface proteins; H44/76, factor H binding protein; hSBA, human serum bactericidal antibody; MenB, *Neisseria meningitidis* serotype B; MenB-4C, 4-component protein-based menB; NZ98/254, New Zealand outer membrane vesicle.

**Figure 3:**
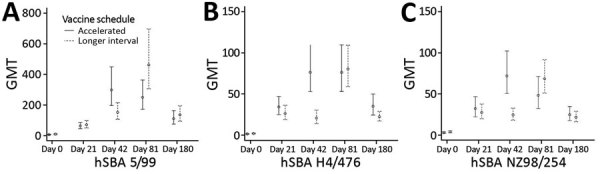
GMTs of hSBA titers to 3 vaccine strains in recipients in trial of 4-component protein-based meningococcal B vaccine administered at 0 and 21 days compared with 0 and 60 days, Canada. A) hSBA 5/99; B) hSBA H44/76; C) hSBA 982/54. Error bars indicate 95% CIs. GMT, geometric mean titer; hSBA, human serum bactericidal antibody; hSBA 5/99, Neisserial adhesin A surface proteins; hSBA H44/76, factor H binding protein; hSBA 982/54, New Zealand outer membrane vesicle.

GMTs increased up to 22-fold from baseline 21 days after the first dose of MenB-4C (Table 3). At day 42, GMTs to the NZ 98/254 and the H44/76 strains were much higher for participants in the accelerated schedule group than for those in the longer interval, but both groups had similar GMTs to all 3 strains thereafter (Table 3; Figure 3).

**Table 3:** Geometric mean titers of human serum bactericidal antibody to meningococcal B strains in recipients of a longer interval dosing schedule compared with an accelerated dosing schedule in a trial of MenB-4C vaccine, from day 0 to 180 postvaccine, Canada*

MenB strain	Geometric mean titers (95% CI)				
	Day 0	Day 21	Day 42	Day 81	Day 180
5/99					
Accelerated	5.86 (4.03–8.54)	63.24 (45.87–87.19)	310.99 (207.74–465.55)	262.30 (183.72–374.50)	114.73 (79.32–165.95)
Longer interval	9.34 (6.78–12.87)	74.76 (52.86–105.72)	162.56 (114.85–230.09)	482.30 (15.61–737.02)	144.55 (99.88–209.2)
H44/76					
Accelerated	1.50 (1.25–1.80)	34.38 (24.93–47.42)	79.66 (54.86–115.67)	77.75 (54.02– 111.90)	35.7 (24.8– 51.39)
Longer interval	2.10 (1.66– 2.66)	27.40 (19.74–38.01)	22.9 (15.68–33.45)	85.26 (62.47–116.37)	23.33 (17.94–3.33)
NZ98/254					
Accelerated	3.08 (2.29–413)	32.38 (22.19–47.25)	75.88 (53.30–108.02)	48.98 (33.12–72.43)	25.71 (18.38–35.96)
Longer interval	4.05 (3.00–5.47)	28.06 (20.28–38.82)	25.81 (19.35–34.42)	69.58 (51.27–94.45)	22.22 (16.41–30.09)

*Geometric mean titers of serum bactericidal antibody using human serum as a complement source. 5/99, Neisserial adhesin A surface proteins; H44/76, factor H binding protein; hSBA, human serum bactericidal antibody; MenB, *Neisseria meningitidis* serotype B; MenB-4C, 4-component protein-based menB; NZ98/254, New Zealand outer membrane vesicle.

We compared the percentage of participants achieving an hSBA titer >1:4 to the 3 MenB-4C strains in the accelerated schedule group against the longer interval group at each study visit (Appendix Table 2). We found the schedules were comparable at all points to the longer interval after vaccination.

### Reactogenicity

**Figure 4:**
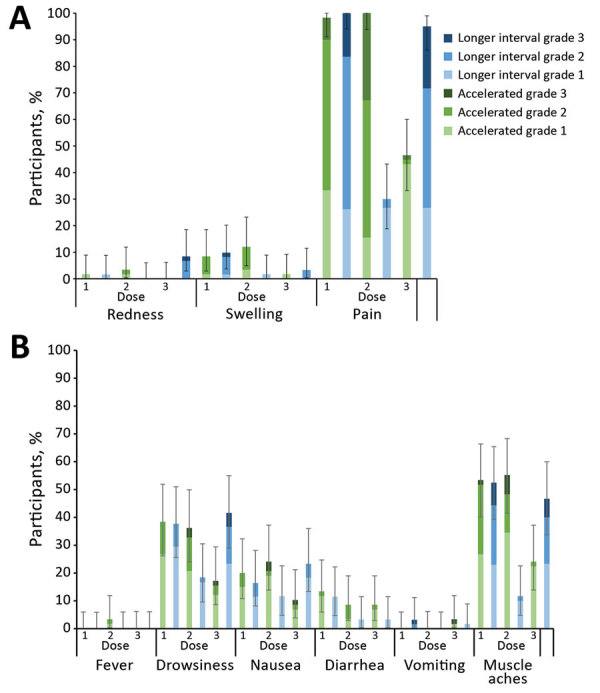
Percent of participants reporting solicited local and systemic adverse events on day 0 and day 8 after each vaccine dose in trial of 4-component protein-based meningococcal B vaccine, Canada. A-C) Adverse events localized at injection site. D-F) General adverse events. Grade 1: mild, easily tolerated by participant; grade 2: moderate, sufficiently discomforting to interfere with normal everyday activities; grade 3: severe, prevents normal, everyday activities. Error bars indicate 95% CIs.

The most common solicited injection site AE was pain, which occurred in 95%−100% of participants after each dose of MenB-4C, and 8.3–32.8% of participants rated their pain as grade 3, interferes with normal activity (Figure 4, panel A). Of participants receiving HAV, 30%–46.6% reported injection site pain; none reported grade 3 pain. The most common systemic AE was muscle aches, reported by 46.7%–55.2% of MenB-4C recipients and 11.7%–24.1% of HAV recipients. In the accelerated schedule group, 2 participants had fever after the second dose of MenB-4C (Figure 4, panel B). The rate of unsolicited AEs did not differ by dosing schedule or dose (data not shown). One participant had a serious AE (fractured patella) unrelated to vaccination.

## Discussion

Invasive meningococcal disease outbreaks cause high levels of distress about the risk for secondary cases from common affiliation in the organization or by a shared, geographically defined community where the outbreak is occurring (*3*). Investigating and managing suspected outbreaks is a time-consuming and resource-intensive effort (*14*). When resources are directed to a mass vaccination program, other public health or healthcare delivery activities might be suspended. Mass vaccination programs in educational settings require planning around midterm and final examinations, school breaks, and the academic year. Immunization programs that rely on multiple doses of vaccine given months apart are even more challenging. 

A variety of MenB-4C dosing intervals, from 30–70 days, have been used to control meningococcal outbreaks on college campuses since the vaccine’s licensure. Limited data have been reported on the immunogenicity of these schedules among students 17–25 years of age (*13*), and few studies have assessed duration of protection. Shorter dosing schedules can improve compliance and lead to higher vaccine coverage (*11*). An accelerated dosing schedule for MenB-4C vaccine in a campus setting would reduce the time required to conduct the vaccine campaign and the resources required. 

We conducted a randomized, controlled, multicenter study in students 17–25 years of age in Canada to assess an accelerated MenB-4C schedule of 2 doses administered 21 days apart. We found the accelerated schedule produced high immunogenicity comparable to 2 doses administered 60 days apart. We observed that >95% of participants in the accelerated and the longer interval groups had protective antibody responses at day 180. We did not observe an increase in AEs with the accelerated schedule, and the most frequent AEs were injection site pain and muscle aches, which were more common after MenB-4C than the control vaccine regardless of the vaccine schedule. 

Baseline protective titers to the 3 MenB vaccine components among participants ranged from 20.7% to 89.9%. Naturally acquired serum antibodies can occur after asymptomatic nasopharyngeal carriage of pathogenic and nonpathogenic *Neisseria* strains, and cross-reacting antibodies might be elicited to non-*Neisseria* strains in the gut flora (*20*). Beginning in 2002, children in Canada have received an oligosaccharide meningococcal C protein conjugate vaccine (MenC) at 12 months of age. Provincial and territorial programs in Canada provide a MenC vaccine or a quadrivalent MenC-ACYW-135 vaccine to adolescents. The participants in our study likely would have had exposure only to a MenC vaccine. The possibility that exposure to either of these vaccines can induce cross-reactive antibodies to MenB-4C vaccine components is unknown.

Although the approved schedule for MenB-4C vaccine is 2 doses, we noted a rapid and robust immune response to the first dose of MenB-4C vaccine in our study group by day 21. We hypothesize that a single dose could be considered for the purpose of outbreak management in persons 17–25 years of age when short-term protection is needed during an academic year. No direct evidence has been reported on the duration of individual protection against invasive meningococcal disease in this age group after a single dose of MenB-4C vaccine. In a trial of children 11–17 years of age randomized to different schedules, investigators noted protective titers >4 in 69%–81% of 1-dose participants at 6 months (*18*). In that study, only 57% (95% CI 49%–65%) of participants who did not have protective titers at baseline had protective titers at 6 months. 

Coverage for MenB in university-based mass immunization campaigns in the United States during 2013–2018 was variable; 14%–98% of the population received the first dose (*4*), and <50% of the population returned for the second dose in half of those campaigns. If future research demonstrates acceptable short-term protection and outbreak control with a single-dose strategy, resource and opportunity cost savings could allow investigators to focus on increasing coverage rates for the first dose.

Our study had some limitations. First, we assessed immunogenicity to MenB-4C vaccine by using hSBA responses to 3 reference strains, but these strains might differ in antigen expression from diverse circulating strains (*21*). However, even if responses to other strains had been tested, negative hSBA results do not necessarily indicate susceptibility to disease because the assay underestimates immunity (*22*–*24*). Second, we did not evaluate hSBA responses beyond 180 days, so the durability of protection beyond the study period and into the subsequent academic year is unknown.

In summary, we conducted a randomized, controlled, observer-blinded multicenter trial of MenB-4C vaccination in students 17–25 years of age. We demonstrated that an accelerated immunization schedule of 0 and 21 days had comparable immunogenicity to a longer interval schedule of 0 and 60 days. We also observed that protective hSBA titers were sustained for >6 months. An accelerated vaccine schedule could be considered to control outbreaks, would be easier to schedule within the constraints of the academic year, and can optimize public health and campus resources by deploying human resources to implement the program in a condensed period. Our data support the use of an accelerated MenB-4C vaccine schedule of 2 doses given at 0 and 21 days to rapidly control meningococcal disease outbreaks on college campuses.

Appendix Additional information on a randomized trial of 2 meningococcal B vaccine schedules in adolescents and young adults, Canada.
