# Supplementary material for: Randomized Trial of 2 Schedules of Meningococcal B Vaccine in Adolescents and Young Adults, Canada
Source: Emerg Infect Dis. 2020 Mar;26(3):454–62. doi: 10.3201/eid2603.190160 (PMC7045834; doi:10.3201/eid2603.190160)
Supplement: Appendix — Additional information on a randomized trial of 2 meningococcal B vaccine schedules in adolescents and young adults, Canada. [file 19-0160-Techapp-s1.pdf]

Article DOI: https://doi.org/10.3201/eid2603.190160

Randomized Trial of 2 Schedules of Meningococcal B Vaccine in Adolescents and Young Adults, Canada

Appendix

Appendix Table 1. Adverse event severity grading for randomized trial of 2 schedules of meningococcal B vaccine in adolescents and young adults, Canada

Adverse event and intensity score*	Parameter
Pain at injection site	
0	None
1 Mild	Any pain neither interfering with nor preventing normal every day activities.
2 Moderate	Painful when limb is moved and interferes with every day activities.
3 Severe	Significant pain at rest. Prevents normal every day activities.
Redness or swelling at injection site	Record greatest surface diameter in mm
0	≤20 mm
1 Mild	>20 mm to ≤50 mm
2 Moderate	>50 mm to ≤100 mm
3 Severe	>100 mm
Fever†	Record temperature in °C or °F
1	>38.0°C–≤38.5°C (100.4°F–101.3°F)
2	>38.5°C–≤39.0°C (101.3°F–102.2°F)
3	>39.0°C–≤40.0°C (102.2°F–104°F)
4	>40.0°C (104°F)
Drowsiness; generalized muscle aches; or gastrointestinal symptoms (e.g., nausea, vomiting, or diarrhea)	
0	No reported symptoms
1 Mild	An adverse event that is easily tolerated by the participant, causes minimal discomfort, and does not interfere with everyday activities
2 Moderate	An adverse event that is sufficiently discomforting to interfere with normal everyday activities
3 Severe	An adverse event that prevents normal everyday activities

*Unsolicited adverse events were assigned to the categories of mild, moderate, or severe at the discretion of the investigator.
†Fever was defined as temperature >38.0°C or >100.4°F for oral, axillary, or tympanic route; the preferred route was oral.

Appendix Table 2. Percentage of participants with human serum bactericidal antibody titers ≥1:4 to 3 strains of meningococcal B at each study visit in a trial of 4-component protein-based meningococcal B (MenB-4C) vaccine, Canada*

Day of study, vaccine group	Sample size	No. (%)	95% CI	p value
5/99 (Neisserial adhesin)				
Day 0				
Accelerated	60	41 (68.3)	57.1–78.2	0.832
Longer interval	61	55 (90.2)	81.5–95.6	
Day 21				
Accelerated	59	59 (100)	95.0–100	0.000
Longer interval	60	59 (98.3)	92.3–99.9	
Day 42				
Accelerated	59	59 (100)	95.0–100	0.000
Longer interval	61	60 (98.4)	92.5–99.9	
Day 81				
Accelerated	59	59 (100)	95.0–100	
Longer interval	61	61 (100)	95.2–100	
Day 180				
Accelerated	59	58 (98.3)	92.2–99.9	0.002
Longer interval	60	60 (100)	95.1–100	
H44/76 (factor H binding protein)				
Day 0				
Accelerated	60	14 (23.3)	14.7–34.0	0.758
Longer interval	61	27 (44.3)	33.4–55.6	
Day 21				
Accelerated	59	58 (98.3)	92.2–99.9	0.001
Longer interval	60	59 (98.3)	92.3–99.9	
Day 42				
Accelerated	59	58 (98.3)	92.2–99.9	0.000
Longer interval	61	57 (93.4)	85.6–97.7	
Day 81				
Accelerated	59	58 (98.3)	92.2–99.9	0.002
Longer interval	61	61 (100)	95.2–100	
Day 180				
Accelerated	59	57 (96.6)	89.7–99.4	0.006
Longer interval	60	60 (100)	95.1–100	
982/54 (New Zealand outer membrane vesicle)				
Day 0				
Accelerated	60	31 (51.7)	40.3–62.9	0.525
Longer interval	61	41 (67.2)	56.0–77.1	
Day 21				
Accelerated	59	58 (98.3)	92.2–99.9	0.002
Longer interval	60	60 (100)	95.1–100	
Day 42				
Accelerated	59	59 (100)	95.0–100	0.000
Longer interval	61	60 (98.4)	92.5–99.9	
Day 81				
Accelerated	59	59 (100)	95.0–100	
Longer interval	61	61 (100)	95.2–100	
Day 180				
Accelerated	59	57 (96.6)	89.7–99.4	0.003
Longer interval	60	59 (98.3)	92.3–99.9	

*The accelerated schedule was MenB-4C vaccine at 0 and 21 days. The longer interval schedule was MenB-4C vaccine at 0 and 60 days
